# Effect of alpha-lipoic acid on radiation-induced small intestine injury in mice

**DOI:** 10.18632/oncotarget.7874

**Published:** 2016-03-03

**Authors:** Bae Kwon Jeong, Jin Ho Song, Hojin Jeong, Hoon Sik Choi, Jung Hwa Jung, Jong Ryeal Hahm, Seung Hoon Woo, Myeong Hee Jung, Bong-Hoi Choi, Jin Hyun Kim, Ki Mun Kang

**Affiliations:** ^1^ Department of Radiation Oncology, Gyeongsang National University School of Medicine and Gyeongsang National University Hospital, Jinju, Republic of Korea; ^2^ Department of Internal Medicine, Gyeongsang National University School of Medicine and Gyeongsang National University Hospital, Jinju, Republic of Korea; ^3^ Department of Otolaryngology, Gyeongsang National University School of Medicine and Gyeongsang National University Hospital, Jinju, Republic of Korea; ^4^ Institute of Health Science, Gyeongsang National University, Jinju, Republic of Korea; ^5^ Biomedical Research Institute, Gyeongsang National University Hospital, Jinju, Republic of Korea; ^6^ Department of Nuclear Medicine and Molecular Imaging, Gyeongsang National University, Jinju, Republic of Korea

**Keywords:** radiation therapy, alpha-lipoic acid, small intestine, oxidative stress, inflammation

## Abstract

**Purpose:**

Radiation therapy is a highly effective treatment for patients with solid tumors. However, it can cause damage and inflammation in normal tissues. Here, we investigated the effects of alpha-lipoic acid (ALA) as radioprotection agent for the small intestine in a mouse model.

**Materials and Methods:**

Whole abdomen was evenly irradiated with total a dose of 15 Gy. Mice were treated with either ALA (100 mg/kg, intraperitoneal injection [i.p.]) or saline (equal volume, i.p.) the prior to radiation as 100 mg/kg/day for 3 days. Body weight, food intake, histopathology, and biochemical parameters were evaluated.

**Results:**

Significant differences in body weight and food intake were observed between the radiation (RT) and ALA + RT groups. Moreover, the number of crypt cells was higher in the ALA + RT group. Inflammation was decreased and recovery time was shortened in the ALA + RT group compared with the RT group. The levels of inflammation-related factors (i.e., phosphorylated nuclear factor kappa B and matrix metalloproteinase-9) and mitogen-activated protein kinases were significantly decreased in the ALA + RT group compared with those in the RT group.

**Conclusions:**

ALA treatment prior to radiation decreases the severity and duration of radiation-induced enteritis by reducing inflammation, oxidative stress, and cell death.

## INTRODUCTION

Cancer is the leading cause of death in economically developed countries and the second leading cause of death in developing countries in 2011 [1]. Management of cancer is the major goal of oncology physicians and radiation therapy, alone or combined with chemotherapy or surgery, is the standard treatment modality for cancer. The aim of radiation therapy in the management of cancer is to maximize the effects of radiation therapy by increasing the radiation dose to the cancer and concurrently minimize side effects by decreasing the radiation dose to the normal organs, thereby increasing the therapeutic ratio [2]. It is important to improve the patient's quality of life, and survival can be prolonged through effective cancer management by using appropriate and effective radiation therapy.

Enteritis is the most common side effect of radiation therapy in the treatment of abdominal or pelvic cancer, including gastric, pancreatic, rectal, cervical, and endometrial cancers [3], and causes decreased quality of life through poor oral intake and absorption of nutrition. In the small intestine, the tolerance dose, defined as the highest dose of radiation that normal organs can endure, acts as a limiting factor for the radiation dose and is a predictive factor for radiation-induced enteritis. Radiation-induced enteritis is classified into six grades (0–5) depending on its severity [4].

Several methods have been proposed to enhance the efficacy or reduce the side effects of radiation therapy. One method for enhancing the efficacy of radiation therapy is to combine radiation therapy with other treatment modalities, such as surgery or chemotherapy. The purpose of combined therapy is to increase the sensitivity of the tumor to radiation therapy [3, 5]. Alternatively, the therapeutic ratio can be increased using advanced techniques such as image-guided radiation therapy or stereotactic radiosurgery, resulting in an increase in irradiation dose to the target volume without increase in irradiation dose to the normal organs [5]. Moreover, the side effects of radiation therapy can be reduced by conducting high technique of radiation therapy, as mentioned above, and by decreasing the irradiated volume of normal organs [5, 6]. Another method to reduce side effects is to decrease the sensitivity of normal organs to radiation by using radioprotective agents rather than increasing the sensitivity of tumors by using radiation sensitizers. Although many studies have examined the radioprotective effects of various agents [7–10], none has been used widely in clinical settings. Additionally, the ability of such agents to reduce the side effects caused by radiation therapy and the radioprotective effects of the agent on cancer cells are unclear.

Reactive oxygen species (ROS) generated by radiation causes radiation-induced complications, and thus antioxidant compounds have been used to scavenge these free radicals [11–14]. N-acetylcysteine and alpha lipoic acid (ALA), glutathione (GSH)-elevating agents, are typical antioxidant compounds. Both of which are non-toxic within certain concentration ranges in humans [15, 16]. ALA is a strong antioxidant with high reactivity to free radicals and facilitates generation of vitamin C and E to elevate tissue levels of GSH. ALA has been shown to be effective in preventing pathological processes involving ROS, such as ischemia-reperfusion injury, diabetes, hypertension, radiation injury, and human immunodeficiency virus activation [17–21]. Moreover, ALA is known to protect hematopoietic tissue against radiation injury when increasing the median lethal dose (LD50) and dose-reduction factor in mice [22].

Here, we aimed to investigate whether ALA could reduce the occurrence of radiation-induced enteritis in a mouse model.

## RESULTS

### ALA alleviated the clinical symptoms of radiation-induced small intestinal injury

**Figure 1:**
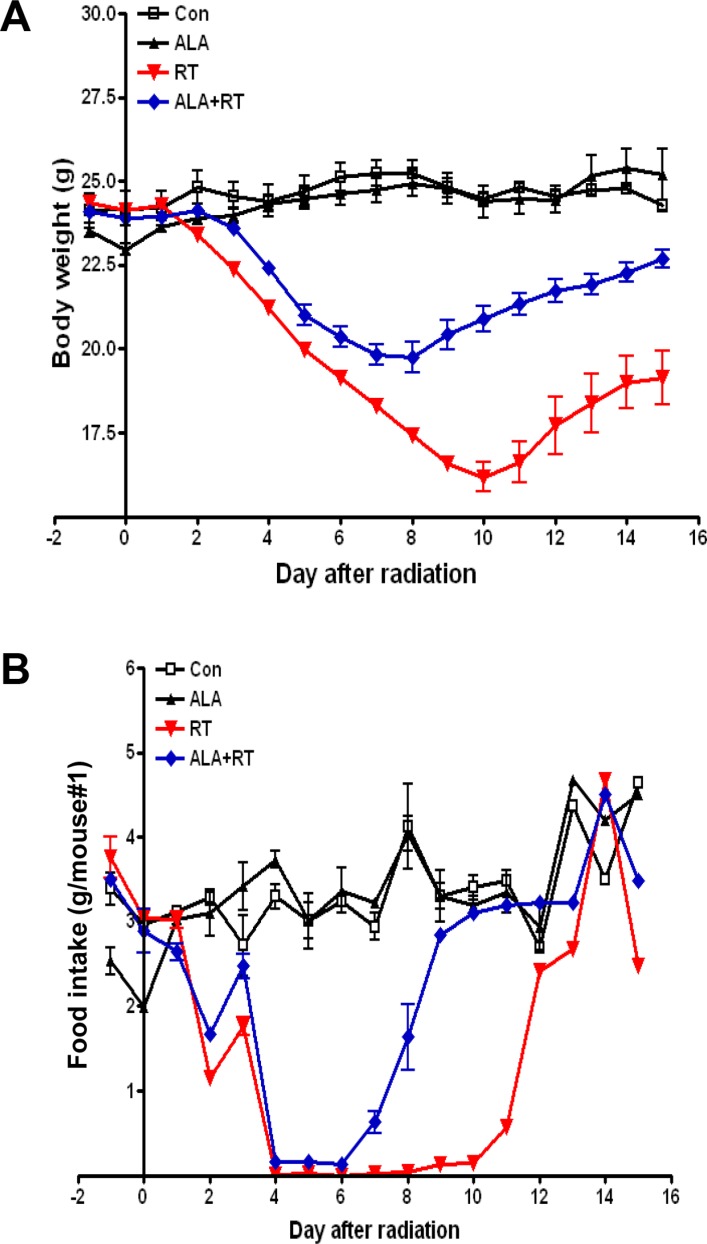
Changes in body weight and food intake in mice with radiation-induced small intestinal injury Body weight and food intake were monitored daily in all mice. All experiments were repeated three times. Data are expressed as the mean ± standard error (SE; *n* = 10 mice/group).

The clinical severity of radiation-induced small intestinal injury was monitored by measuring body weight and food intake daily. No clinically significant weight loss or reduced food intake was observed in the control and ALA groups. However, irradiation induced marked decreases in body weight and food intake 3–10 and 2–10 days after radiation, respectively. The ALA + radiation (RT) group showed a moderate degree of weight loss and improved food intake following irradiation as compared with the RT group (Figure 1), suggesting that ALA may reduce clinical symptoms in the small intestine of mice subjected to radiation.

### ALA protected against radiation-induced small intestinal mucosal injury

**Figure 2:**
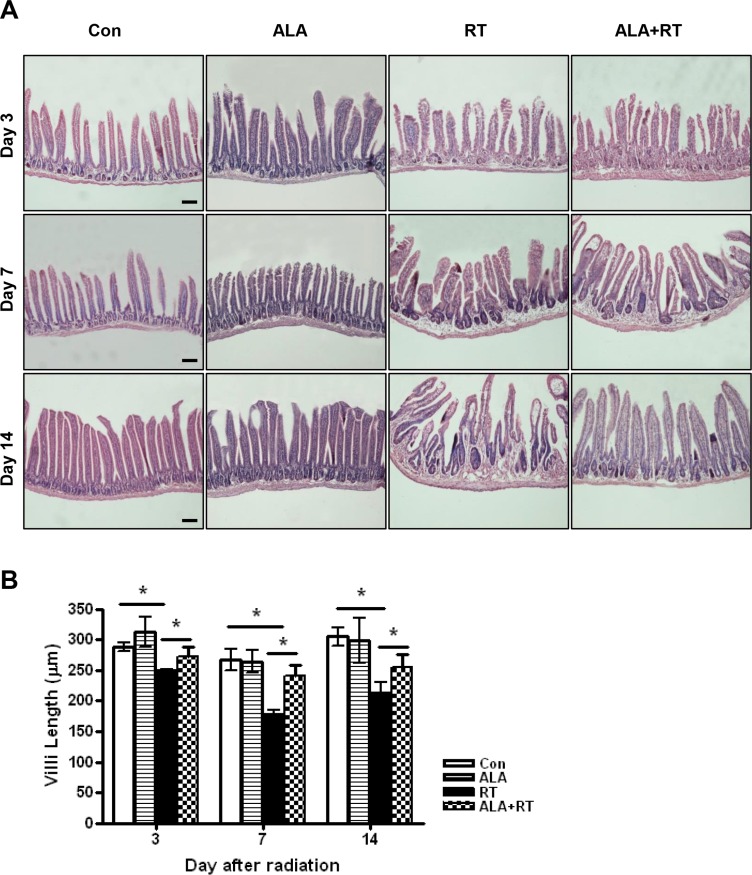
Histopathological changes of the small intestine at 3, 7, and 14 days after irradiation Height measurements from 10 villi were obtained in small intestine sections from each group at 200× magnification. Each bar shows the mean ± SE; *p < 0.05 indicates differences between groups. Con: control group; RT: radiation group; ALA + RT: received alpha-lipoic acid (ALA) before irradiation. Scale bar; 100 μm. Con and ALA (*n* = 4/each day), RT and ALA + RT (*n* = 10/each day).

The height of villi reflects the degree of overall mucosal damage. Thus, we evaluated histological changes by measuring the small intestinal villus height. The control and ALA groups exhibited tall, well-arranged mucosal epithelial cells. However, irradiation caused severe mucosal damage (Figure 2A). Villus height decreased significantly in the RT group compared with the control and this decrease was suppressed in ALA + RT group at all experimental time-points (Figure 2B). Thus, ALA prevented radiation-induced morphological damage in the small intestine.

### ALA decreased apoptosis in the small intestine

**Figure 3:**
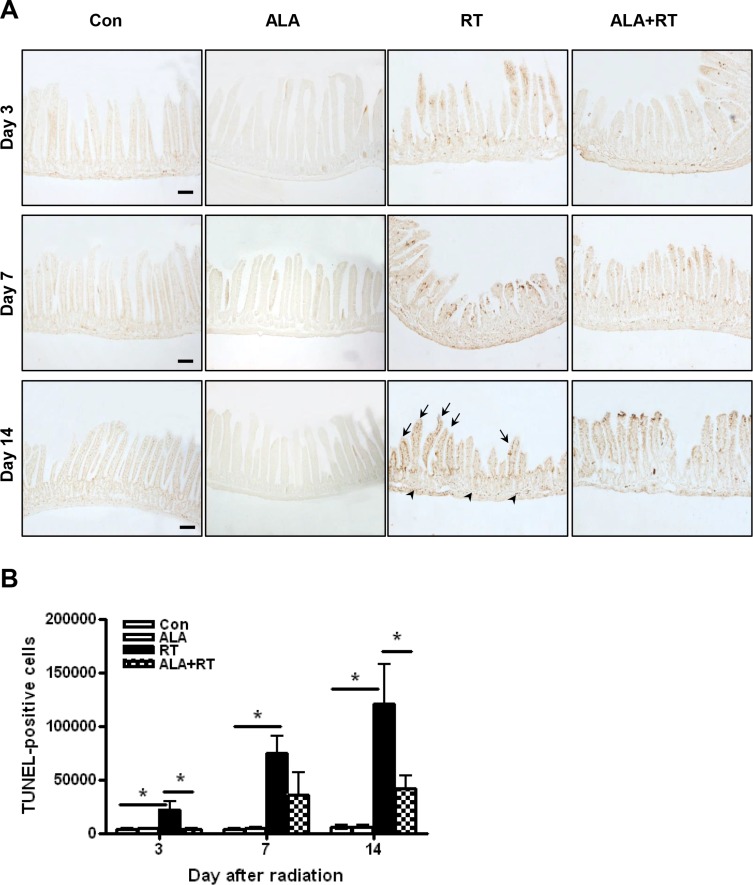
Apoptotic death in the small intestine with radiation-induced small intestinal injury Apoptotic death was defined as the average number and density of terminal deoxynucleotidyl transferase dUTP nick-end labeling (TUNEL)-positive cells in 10 random fields from each section at 400× magnification (**A**). Signals density for TUNEL-positive cells was measured in the marked areas (arrow, edge of the villi. arrowhead, the muscularis mucosa). Each bar represents the mean ± standard error (SE); *p < 0.05 indicates differences between the radiation (RT) and alpha-lipoic acid (ALA) + RT groups (**B**). Con: control group; RT: radiation group; ALA + RT: received ALA before irradiation. Scale bar; 100 μm. Con and ALA (*n* = 4/each day), RT and ALA + RT (*n* = 10/each day).

Apoptosis is a major pathogenic feature of radiation-induced small intestinal mucosal injury, and the degree of apoptosis reflects the degree of mucositis [2]. The degree of apoptosis was assessed using terminal deoxynucleotidyl transferase dUTP nick-end labeling (TUNEL) assay. TUNEL-positive signals in the small intestine significantly increased in the RT group compared with the control and ALA groups at each time point (Figure 3A). Most positive signals were detected at the edge of villi in the small intestinal mucosa. However, mice in the ALA + RT group exhibited a significant decrease in radiation-induced TUNEL-positive cells compared with the RT group (Figure 3B). These data indicate that ALA protected the small intestine against radiation-induced apoptosis.

### ALA restored GSH levels and reduced oxidative stress following radiation

**Figure 4:**
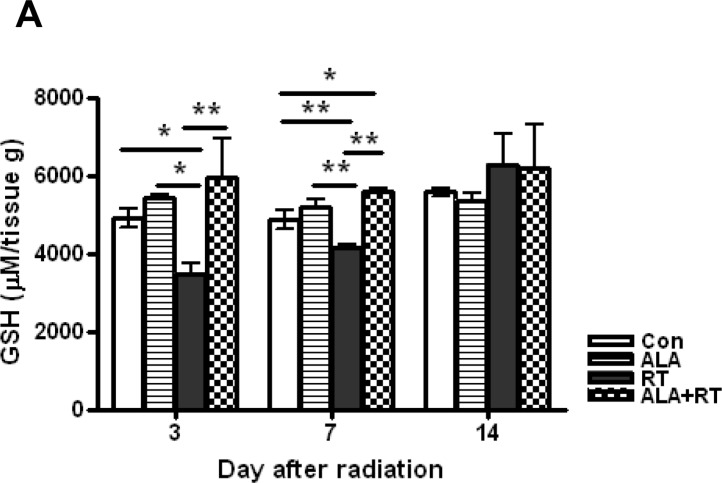

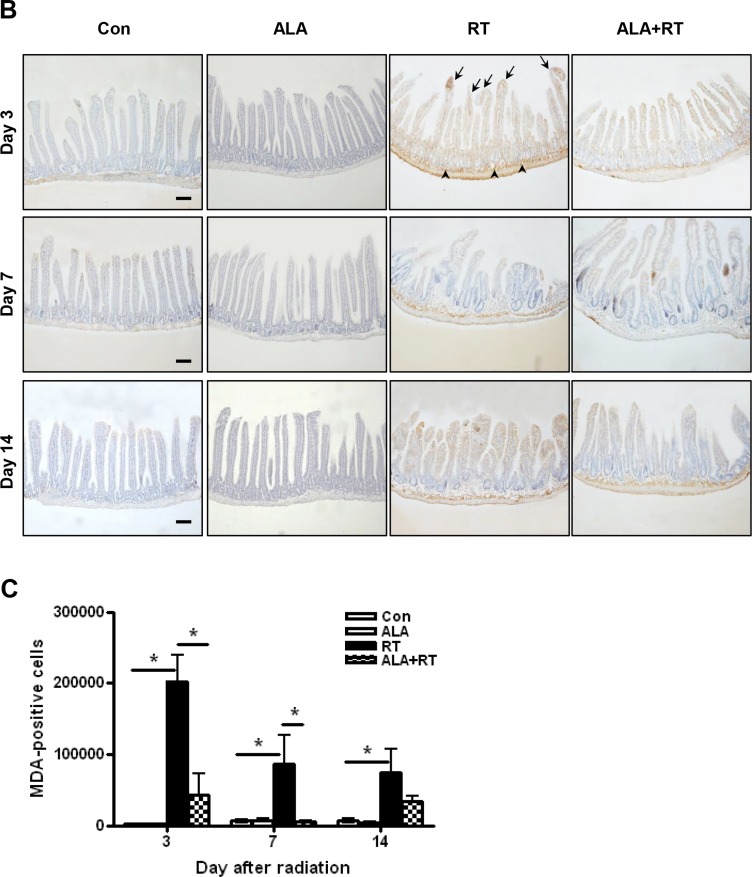

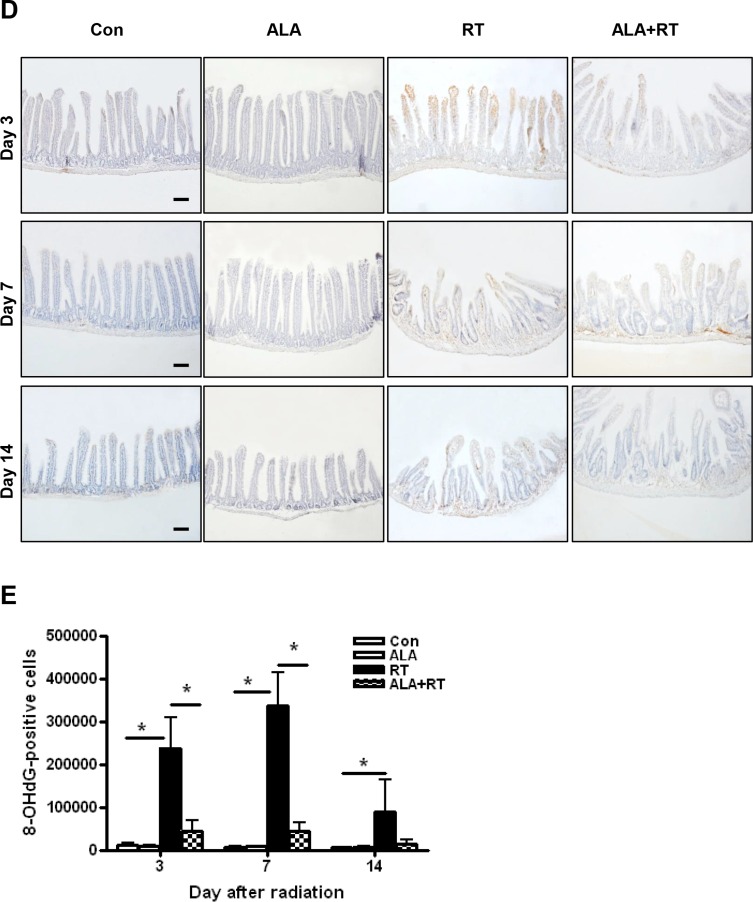
Oxidative stress induced by radiation Small intestinal tissue glutathione (GSH) levels were measured. Data are the mean ± standard error (SE); *p < 0.05 and **p < 0.05 indicate differences between groups (**A**). Malondialdehyde (MDA) was predominantly expressed at the edges of villi and in the muscularis externa and serosa (**B**). 8-Hydroxy-2′-deoxyguanosine (8-OHdG)-positive cells were localized at the small intestinal mucosa and submucosa and the muscularis externa and serosa (**D**). Signals density for MDA and 8-OHdG-positive cells were measured in the marked areas (arrow, edge of the villi. arrowhead, the muscularis mucosa). Data are the mean ± standard error (SE); *p < 0.05 indicate differences between groups (**C** and **E**). Scale bar; 100 μm. Con: control group; RT: radiation group; ALA + RT: received alpha-lipoic acid (ALA) before irradiation. Con and ALA (*n* = 4/each day), RT and ALA + RT (*n* = 10/each day).

Tissue GSH levels decreased significantly in the RT group at 3 and 7 days after radiation. In the ALA + RT group, a significant increase was noted in tissue GSH levels compared with the RT group at 3 and 7 days after radiation; however, this difference was not significant at 14 days after radiation (Figure 4A). Ionizing radiation enhances the production of ROS, thereby inducing oxidative damage, including lipid peroxidation. Malondialdehyde (MDA) is a representative marker of lipid peroxidation. Therefore, we examined MDA expression in the small intestine of mice in the four groups. MDA-positive signals were mainly detected at the edges of villi and in the muscularis externa and serosa (Figure 4B). Radiation significantly increased the MDA-positive signals in the small intestine. ALA administration significantly decreased MDA expression induced by radiation at days 3 and 7 after radiation but the decrease was not significant at day 14, similar to that observed for tissue GSH levels (Figure 4C). Additionally, immunohistochemical staining of 8-hydroxy-2′-deoxyguanosine (8-OHdG), a ROS-induced DNA damage marker, was performed to investigate the effects of ALA on radiation-induced oxidative stress (Figure 4D). 8-OHdG-positive cells were detected at the edges of the villi, significantly increased on day 3 and peaked on day 7 after radiation, and these increases were remarkably suppressed in ALT + RT group (Figure 4E). Similar to the tissue GHS level and MDA expression, 8-OHdG expression did not significant alter at day 14 after radiation. These data suggest that ALA may reduce oxidative stress at the acute phase of radiation.

### ALA decreased MMP-9 expression, NF-κB phosphorylation, and serum IL-1β and IL-6 levels

**Figure 5:**
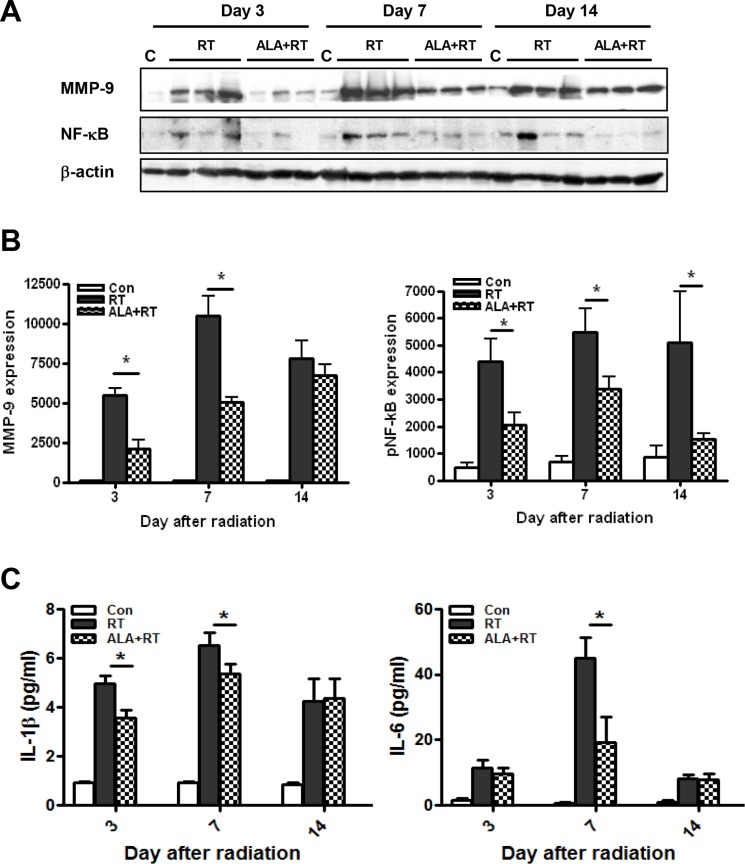
Western blotting was performed using anti-MMP-9 and anti-phosphorylated NF-κB antibodies β-actin was evaluated as the loading control (**A**). Alpha-lipoic acid (ALA) inhibited radiation-induced expression of MMP-9 and phosphorylated of NF-κB in the RT-induced small intestine. Con (*n* = 3/each day), RT and ALA + RT (*n* = 9/each day, *n* = 3/each lane) (**B**). ELISA analysis of serum IL-1β and IL-6 levels from each group on each day. Con (*n* = 5/each day), RT and ALA + RT (*n* = 10/each day and group) (**C**). Data are the mean ± standard error (SE); *p < 0.05 indicate differences between groups. Con: control group; RT: radiation group; ALA + RT: received ALA before irradiation.

**Figure 6:**
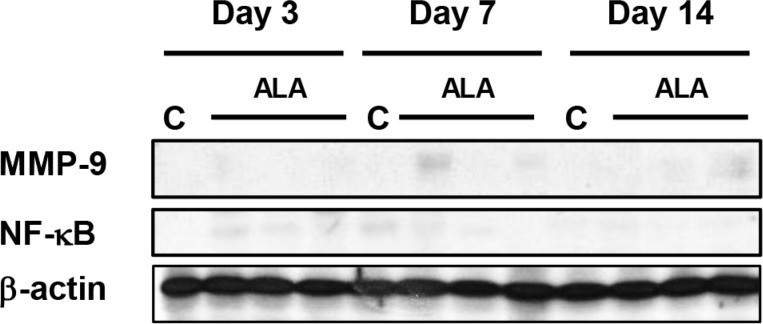
Western blotting was performed using anti-MMP-9 and anti-phosphorylated NF-κB antibodies β-actin was evaluated as the loading control. Con: control group, ALA: alpha-lipoic acid (ALA) alone group. Con and ALA (*n* = 3/each day).

Radiation-induced enteritis is characterized by a defined pattern of inflammation and fibrosis, and MMP-9 is involved in the inflammatory response [23]. We found marked induction of MMP-9 expression in irradiated small intestine after 3, 7, and 14 days (Figure 5A). MMP-9 expression peaked on day 7, and ALA reduced MMP-9 expression at 3 and 7 days after radiation. The difference was not significant at 14 days after radiation (Figure 5B). In addition to MMP-9, the NF-κB signaling pathway is another major source of inflammation in radiation-induced small intestinal injury [24]. Thus, we next evaluated the activation of NF-κB. Marked induction of phosphorylated NF-κB protein was observed in irradiated small intestinal tissues after 3, 7, and 14 days, peaking at day 7 after irradiation and with high levels maintained until day 14 (Figure 5A). ALA treatment reduced the phosphorylation of NF-κB at all-time points (Figure 5B). We also found that IL-1β and IL-6, two main inflammatory cytokines, increased significantly in irradiated mice and the levels were ameliorated by ALA treatment on day 7 after radiation. In IL-1β, the level is also significantly decreased by ALA on day 3 after radiation (Figure 5C). No significant differences found between control and ALA alone group in serum IL-1β and IL-6 (data not shown). No significant expression differences were observed for MMP-9 and NF-κB in the control and ALA groups (Figure 6). These data suggest that ALA decreased inflammation in irradiated small intestinal tissues.

## DISCUSSION

Radiation therapy is one of the primary modalities for treating patients with cancers of the pelvic cavity or abdomen [25–28]. Enteritis is the most common side effect of radiation therapy for the treatment of abdominal cancer, and the susceptibility of the small intestine to radiation-induced damage is the limiting factor for the prescription dose of radiation therapy. Enteritis caused by radiation therapy, which is accompanied by various clinical symptoms, reduces the patient's quality of life and increases the cost of treatment and the social health care cost [29, 30]. Currently, to our knowledge, there are no widely used methods for reducing the occurrence or severity of radiation-induced enteritis. We aimed to investigate the effect of ALA in the prevention or treatment of radiation-induced enteritis. Our results showed that administration of ALA prior to radiation significantly alleviated the clinical symptoms of radiation-induced enteritis, including reduced food intake and weight loss. These effects may be explained by reductions in cell death, oxidative stress, and inflammation.

Our results showed reductions in food intake and weight loss similar to those in patients with pelvic or abdominal cancer who underwent radiation therapy. These symptoms were less serious and recovered more rapidly in the ALA-treated group. The observed weight loss may have resulted from poor food intake caused by shortening of the villi after radiation. Tissue damage, which began in the submucosal area and migrated to the epithelium, was lower in the ALA + RT group than in the RT group. The height of small intestinal villi were greater in the ALA + RT group than in the RT group. Thus, these data showed that ALA could attenuate radiation-induced bowel damage. The migration of tissue damage was in line with the traditional concept that radiation-induced tissue damage is more rapid and severe in rapidly proliferating tissue [2].

**Table 1:** Experimental design and time table for treatment

Days																	
	2	1	0	1	2	3	4	5	6	7	8	9	10	11	12	13	14
ALA	√	√	√														
Radiation			√														
Sampling						√				√							√

Thirty-nine BALB/c mice were randomized to four groups: i) control group (9 mice), ii) ALA group (9 mice), iii) RT group (11 mice), and RT + ALA group (10 mice). Small intestine damage was induced by irradiation (15 Gy, 1 fraction) on the abdomen. In the ALA group, mice received 100 mg/kg/day ALA by intraperitoneal injection on days −2 to 0. The control group received vehicle in the same way. The small intestine was collected 3rd, 7th, and 14th days after irradiation.

The GSH level was measured to verify the role of ALA in oxidative stress, which disrupts DNA damage and normal cellular signaling and is involved in various clinical diseases, particularly cancer [31–33]. The observed temporal changes in GSH levels in the RT group supported a normal response to radiation. GSH levels were significantly lower in the RT group than in the ALA + RT group, because antioxidants were consumed to reduce radiation-induced oxidative stress. GSH levels increased by internal homeostasis mechanisms on day 14 after irradiation, although no differences were noted between the RT and ALA + RT groups.

The mechanism by which radiation eliminates cancer cells involves the production of free radicals inside the body and destruction of the DNA structure of cancer cells, leading to cell death [2]. However, in clinical practice, this phenomenon occurs not only in cancer cells but also in normal tissues affected by radiation. Preventing DNA damage and subsequent cell death in the normal tissues can reduce the side effects of radiation. To determine the potential differences in level of cell death caused by ALA, we used 8-OHdG as a marker of DNA damage induced by ROS [34, 35], and performed TUNEL assay to estimate cellular apoptosis [36]. 8-OHdG staining showed that DNA damage was inhibited by ALA, consistent with the observed reduced morphological damage in the ALA + RT group. Moreover, we also observed marked differences in apoptosis between the RT and RT + ALA groups. The tendency for increased apoptosis in the RT group may have been caused by mitotic catastrophe, in which apoptosis occurred in cells with DNA damage during the following meiosis. The changes in the localization of the signal over time for both 8-OHdG and TUNEL staining suggest migration from the submucosa to epithelium, as supported by hematoxylin and eosin (H & E) staining. Radiation-induced cell membrane damage, as measured by MDA staining [37], also reduced due to ALA. These results suggest that pretreatment with ALA before radiation could reduce cellular DNA damage or membrane damage induced by free radicals and ultimately reduce the side effects of radiation by preventing cell death in normal tissues.

Inflammation that occurs after irradiation can be induced by a series of processes, including ROS, DNA damage, lipid peroxidation, and apoptosis. Although inflammation is necessary for resolving various types of damage in the body, inflammation occurring after radiation causes various clinical symptoms, and continuous inflammation may result in severe consequences such as cancer. Therefore, resolving inflammation as early is generally beneficial. We evaluated the activation of NF-κB, which plays a crucial role in the immune response against inflammation induced by various stimuli [24], and the expression of MMP-9 [23], which is regulated by NF-κB and is involved in inflammation, tissue repair, and tissue remodeling. Although the pattern of MMP-9 expression in response to radiation was similar to that of NF-κB activation over time, MMP-9 expression in the ALA + RT group tended to increase continuously with time, suggesting that MMP-9 is involved in tissue remodeling, which is associated with fibrosis, a long-term side effect of radiation subsequent to inflammation.

Here, the changes in the small intestine induced by radiation may have been caused by ROS-dependent oxidative stress, triggering DNA and tissue damage and inflammation. It is likely that the apoptosis induced by DNA damage leads to cell death. Inflammation would then occur continuously through factors involved in tissue damage or recovery. In this presumed series of processes, we found that ALA reduced tissue damage, apoptosis, oxidative stress, and inflammation, and as a result, positive outcomes were observed in terms of changes in body weight and food intake.

Although we describe the protective effect of ALA against radiation in the current study, further studies are required to determine the mechanism underlying the effect of ALA. Moreover, we have not yet determined whether the protective effect of ALA against radiation also occurs in cancer cells targeted by radiation. To establish clinical applicability, it will be necessary to determine the differential effects of ALA in normal tissues and cancer cells.

In conclusion, ALA treatment prior to radiation decreased the severity and duration of radiation-induced enteritis by reducing tissue damage, cell death, oxidative stress, and inflammation. Thus, ALA has radioprotective effects against radiation-induced enteritis.

## MATERIALS AND METHODS

### Animals

Male BALB/c mice (7–8 weeks age) were obtained from Koatech Animal Inc. (Peongtaek, Korea). The mice were housed under specific pathogen-free conditions at four animals per polycarbonate cage, with free access to standard hamster food and water, in a room maintained at 23 ± 1°C and 55 ± 5% relative humidity, and a 12 h light/dark cycle (150–300 l×, with lights on from 06:00 to 18:00). All experiments were performed in compliance with institutional guidelines set by the Institutional Animal Care and Use Committee at the Gyeongsang National University (GLA-130621-M0039).

### Experimental design

Forty BALB/c mice were randomized to four groups of 10 mice each, namely, the normal control ALA, RT, and RT + ALA groups. Small intestine damage was induced by irradiation to the abdomen. ALA was administered by intraperitoneal injection (i.p.) three times (100 mg/kg/day, days −2 to 0). Mice in the vehicle group received normal saline administered in the same manner. Blood samples were obtained via direct ventricular puncture, and the small intestine was collected at 3, 7, and 14 days after abdominal irradiation. Experiments were performed three times, independently.

### Radiation exposure

Mice were anesthetized to immobilize them prior to radiation exposure. The whole abdomen was evenly irradiated with 2 Gy/min (total dose of 15 Gy) by using a photon 6-MV linear accelerator (21EX, Varian, Palo Alto, CA, USA). A 1.0-cm-tissue equivalent bolus was positioned on the abdomen to provide adequate buildup. Each mouse was exposed to a single dose of radiation.

### Histopathology

Tissues were fixed in 4% paraformaldehyde in 0.1 M PBS, embedded in paraffin, and cut into 5-μm-thick sections. The sections were stained with H & E.

### Small intestinal histology: height of villi

Segments of the ileum were collected, and the villus height (from the top of the villus to the villus-crypt junction) was measured by light microscopy using a calibrated micrometer (200×). Ten intact and well-oriented villi and crypts were measured and averaged for each sample.

### TUNEL assay

The degree of apoptosis was assessed using TUNEL assay. DNA fragmentation was detected using a kit from Roche Applied Sciences (Indianapolis, IN, USA). Semiquantitative analysis was performed by counting the TUNEL-positive cells per field in the small intestinal tissue at 400× magnification. At least 10 areas in the cortex per slide were selected randomly. The mean number of brown cells in these selected fields was considered to be the number of TUNEL-positive cells. TUNEL-positive signals were analyzed by a blinded observer using NIS Elements BR3.1 (Nikon, Japan) software in 10 randomly selected fields.

### Measurement of GSH levels

The GSH content in the small intestine was measured using Glutathione Assay kits (Sigma, St. Louis, MO, USA) according to the manufacturer's instructions. Briefly, 100 mg of each tissue was homogenized in 0.5 mL of GSH reaction buffer containing 0.1 mL of 5% sulfosalicylic acid. To generate NADPH, 20 μL of NADPH Generation Mix and 140 μL of GSH reaction buffer were mixed and incubated at room temperature for 10 min. Then, 20 μL of either the GSH standard solution or the sample solution was added, followed by incubation at room temperature for 5–10 min. An additional 20 μL of substrate solution was then added. A microplate reader was used to measure the absorbance at 405 nm (Molecular Devices Corp., Sunnyvale, CA, USA).

### Immunohistochemistry

Immunohistochemical analysis was performed on 5-μm-thick sections of paraformaldehyde-fixed, paraffin-embedded tissue using a Vectastain ABC kit (Vector Laboratories, CA, USA). Sections were blocked with 1% normal goat serum and then treated with anti-rabbit MDA (1:100 dilution, Santa Cruz Biotechnology, Santa Cruz, CA, USA) and anti-rat 8-OHdG (1:100 dilution, eBioscience, San Diego, CA, USA) antibodies at 4°C overnight in a humidified chamber. PBS-washed tissue sections were then incubated for 90 min at room temperature with secondary antibodies. Finally, the sections were incubated with avidin-biotinylated horseradish peroxidase (HRP)-complexes for 60 min at room temperature, rinsed in PBS, and developed using DAB with hydrogen peroxidase. The density and number of MDA- and 8-OHdG-positive signals were analyzed by a blinded observer by using NIS Elements BR3.1 (Nikon, Japan) software in 10 randomly selected fields.

### Immunoblot

Tissues were homogenized in lysis buffer. Proteins (50 μg) were loaded on a sodium dodecyl sulfate-polyacrylamide gel. The blots were probed with polyclonal anti-matrix metalloproteinase (MMP)-9 (Cell Signaling Technology), and anti-phospho-nuclear factor kappaB (NF-κB; Santa Cruz Biotechnology) antibodies at 4°C overnight. Primary antibodies were visualized using secondary antibodies with an ECL kit (Amersham Pharmacia Biotech, Piscataway, NJ, USA).

### Measurement of serum IL-1β and IL-6

Serum levels of IL-1β and IL-6 were quantified using specific enzyme-linked immunosorbent assay kits according to the manufacturer's instructions (R & D system, Minneapolis, MN, USA).

### Statistical analysis

Statistical analysis was conducted using Sigma Plot 7.0 software (SPSS Inc., Chicago, IL, USA). Results are presented as the means ± standard errors of the mean. Differences between groups were assessed using one-way analysis of variance, followed by the Tukey test. *P* ≤ 0.05 was considered significant.
